# Natural Radionuclides in Bottled Mineral Waters Consumed in Turkey and Their Contribution to Radiation Dose

**DOI:** 10.1021/acsomega.2c04087

**Published:** 2022-09-15

**Authors:** Aydan Altıkulaç, Aslı Kurnaz, Şeref Turhan, Metehan Kutucu

**Affiliations:** † Ula Ali Koçman Vocational School, Muğla Sıtkı Koçman University, 48640 Ula, Muğla, Turkey; ‡ Department of Physics, Faculty of Science and Letters, Kastomunu University, 37150 Kastamonu, Turkey; § Department of Physics, Institute of Science, Kastamonu University, 37150 Kastamonu, Turkey

## Abstract

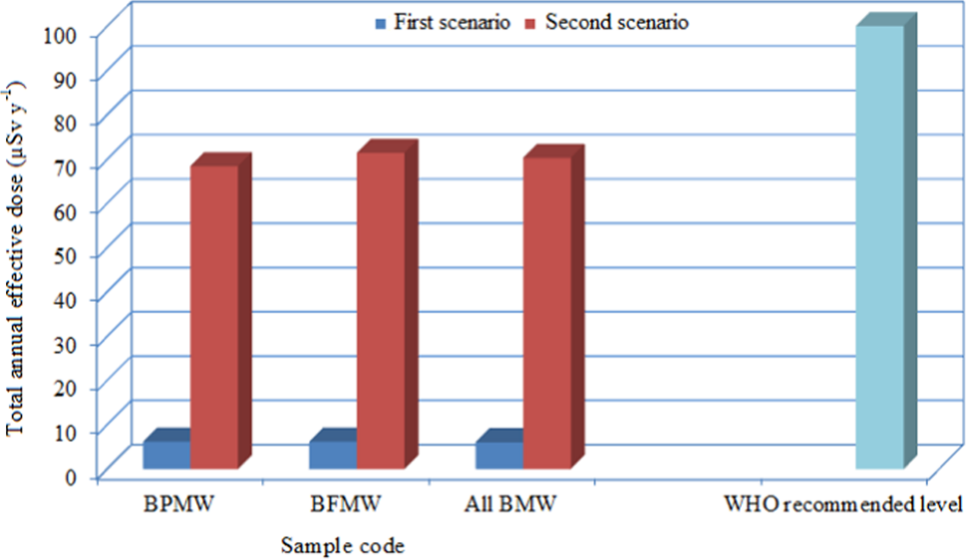

Bottled natural mineral water (BMW) consumption in Turkey is increasing every year. Depending on the local geology from which the water is extracted, BMW could be enhanced with natural radionuclides. In this study, the activity concentrations of natural radionuclides in 58 BMW samples of 25 different brands marketed in Turkey were measured using a γ-ray spectrometer with high-purity germanium (HPGe) detector. The average activity concentrations of ^226^Ra, ^228^Ra, and ^40^K in BMW samples were found as 0.4, 0.5, and 4.3 Bq/L, respectively. The activity concentrations of ^228^Ra exceeded the WHO-recommended maximum permissible limit of 0.1 Bq/L for drinking water. The annual effective dose (AED) and excess lifetime cancer risk (LCR) caused by the ingestion of each BMW sample were estimated for adults to assess radiological risks using two different scenarios based on BMW consumption rates (150 and 13 L/y). All estimated total AEDs, except for two samples, were below the guidance dose level of 100 μSv/y recommended by the World Health Organization (WHO) and Turkish regulations for drinking water. For all BMW brands, ^228^Ra was found as the main contributor to the AEDs. The LCR values were lower than the acceptable value of 10^–3^ for radiological risks.

## 1 Introduction

International organizations such as the Environmental Protection Agency (EPA), International Commission on Radiological Protection (ICRP), United Nations Scientific Committee on the Effects of Atomic Radiation (UNSCEAR), European Union (EU) Council, and World Health Organization (WHO) recommend a daily water intake of at least 1–2 L for adults to avoid health problems.^1,2^ Therefore, the supply of clean, safe, and quality drinking water (tap, spring, mineral, purified, distilled, etc.) is of vital importance. Today, it is becoming one of the most social concerns because water resources (streams, lakes, groundwaters, aquifers, springs, etc.) are vulnerable to contamination with radionuclides, toxic organic and inorganic chemicals, etc. caused by natural events and human activities.^3,4^ Assessment of various water types reveals that groundwater accounts for 99% of freshwater, which is only 2.5% of all water supplies in the world.^5^ It is predicted that about one-third of the world’s population utilizes groundwater as drinking water.^6^ Groundwater contains dissolved minerals and natural radionuclides in the ^238^U and ^232^Th decay series and ^40^K with different concentrations.^7^ The concentrations of these radionuclides depend on the seasonal precipitation variation, the infiltration time, the mineralogical and geochemical composition of the rocks and soil through which the water flows, redox conditions, weathering, exhalation, etc.^8−10^ In some cases, the radionuclide concentrations in groundwater are elevated, and as a consequence, ionizing radiations (α-, β-, and γ-rays) emitted from these ingested and/or inhaled radionuclides pose serious radiological risks to humans.^9,11^ For this reason, the radiological quality of drinking water must be strictly and regularly controlled due to its importance to human health and environmental protection.

Bottled drinking water (BDW) is one of the main ways in which potable water is distributed worldwide, and BDW (mineral and spring) has been promoted worldwide as a more pure, safe, and tastier alternative.^12^ Recently, there has been an increasing trend to replace tap water with bottled mineral water (BMW) due to the importance of BMW in human nutrition and beneficial therapeutic and medical practices.^13^ Turkey has great potential for natural mineral water (NMW) sources and is one of the world’s seven geothermal-potential-rich countries.^13^ However, annual BMW consumption per capita in Turkey is very low when compared to per capita consumption (105 L/y) in European Union (EU) countries.^14^ In the last decade, the popularity and sales volume of BMW have grown rapidly in Turkey after bottled fruit-flavored mineral waters were introduced to Turkish markets. While the annual BMW consumption per capita in Turkey was 6.4 L in 2010, it nearly doubled and reached 13 L in 2021.^15^ According to the Turkish regulations^16^ and EU directives,^17^ NMW must be groundwater (hot or cold) emerging from a spring tapped at one or more natural or bore exits. NMW can be clearly distinguished from ordinary drinking water by its nature, characterized by its mineral content, trace elements, or other constituents, and by its original state. NMW, in its state at the source, may not be the subject of any treatment except for the separation of unstable elements (Fe and S compounds) and elimination or reintroduction of CO_2_.^16,17^ However, the Turkish regulations, EU directives, and WHO guidelines^18^ did not recommend maximum permissible limits (MPLs) for radionuclides in BMWs. The Turkish regulations set the MPLs of 1.5 and 2 Bq/L for gross α and gross β activity concentrations in BMWs.^14^ However, BMW may contain many predominant dissolved natural radionuclides that cause health hazards.^5,19^ Therefore, the radiological quality of mineral water bottled for commercial distribution, whose consumption is increasing year after year in the world, must be carefully and systematically controlled or ensured to be of low radioactivity. When the literature is viewed from this point of view, it is seen that, in recent years, there has been an increased worldwide interest in studies on natural radioactivity measurements in BMWs and extensive studies have been carried out in many countries.^2−5,10,11,20−24^ The available literature shows that there are only a few studies on the determination of activity concentrations of natural radionuclides in Turkish BMWs. Kopya et al.^25^ measured the activity concentrations of ^226^Ra, ^232^Th, ^137^Cs, and ^40^K and gross α/β in 13 mineral water samples collected from six different provinces in the Eastern Black Sea Region of Turkey. Erden et al.^26^ determined the activity concentrations of ^234^U, ^238^U, and ^226^Ra in nine mineral water samples using α-particle spectrometry. Şahin et al.^27^ analyzed the activity concentrations of ^228^Ra in bottled mineral water samples collected from eight different mineral water bottling facilities in Turkey. Seid et al.^13^ determined the activity concentrations of ^222^Rn in 49 BMW samples of 22 commercial brands sold in Turkish markets.

The aim of this study is to obtain detailed information, which is not available in the literature, on the determination of ^226^Ra, ^228^Ra, and ^40^K activity concentrations in BMW samples representing the majority of all BMW brands distributed in Turkish markets and the assessment of radiological risks arising from ingestion of these BMWs because radium isotopes (^226^Ra and ^228^Ra), which accumulate predominantly in bone and soft tissue sarcoma when taken into the body through the digestive tract, are Group A carcinogens.^19,28^ For this aim, in this study, (1) the activity concentrations of ^226^Ra, ^228^Ra, and ^40^K in 58 BMW samples of 25 different best-sold brands consumed in Turkey were measured using a γ-ray spectrometer with an HPGe detector, (2) the radiological risks due to the internal exposure to adults caused by the ingestion of BMW samples were assessed estimating the annual effective dose (AED) and excess lifetime cancer risk (LCR) using two BMW consumption rates, and (3) the measured and estimated values were compared with the maximum permissible limits (MPLs) given in national/international regulations and WHO guidelines for drinking water quality and those obtained for BMWs consumed in other countries.

## 2 Materials and Methods

### 2.1 Collection and Preparation of Samples

Turkey is among the countries rich in mineral waters due to its location in the Alpine-Himalayan geothermal belt, which is one of the most important geothermal belts in the world.^13^ The areas where mineral waters are found in Turkey are generally found in the fracture zones on the edge of Paleozoic massifs. In addition, the fact that the active Quaternary–Upper Tertiary volcanism creates an important heat source is one of the main factors.^29^ NMW areas in Turkey have developed due to the graben structures in the Aegean Region and the Central and Eastern parts of the Anatolian Plate due to the change in frequency due to the effect of neotectonic. There are important geothermal areas in the depths of the North Anatolian Fault Zone and its active opening structures, as well as the sedimentary basins in the Marmara and Southeastern Anatolia regions in the Anatolian Plate, so there are also abundant mineral waters in these areas.

**Figure 1:**
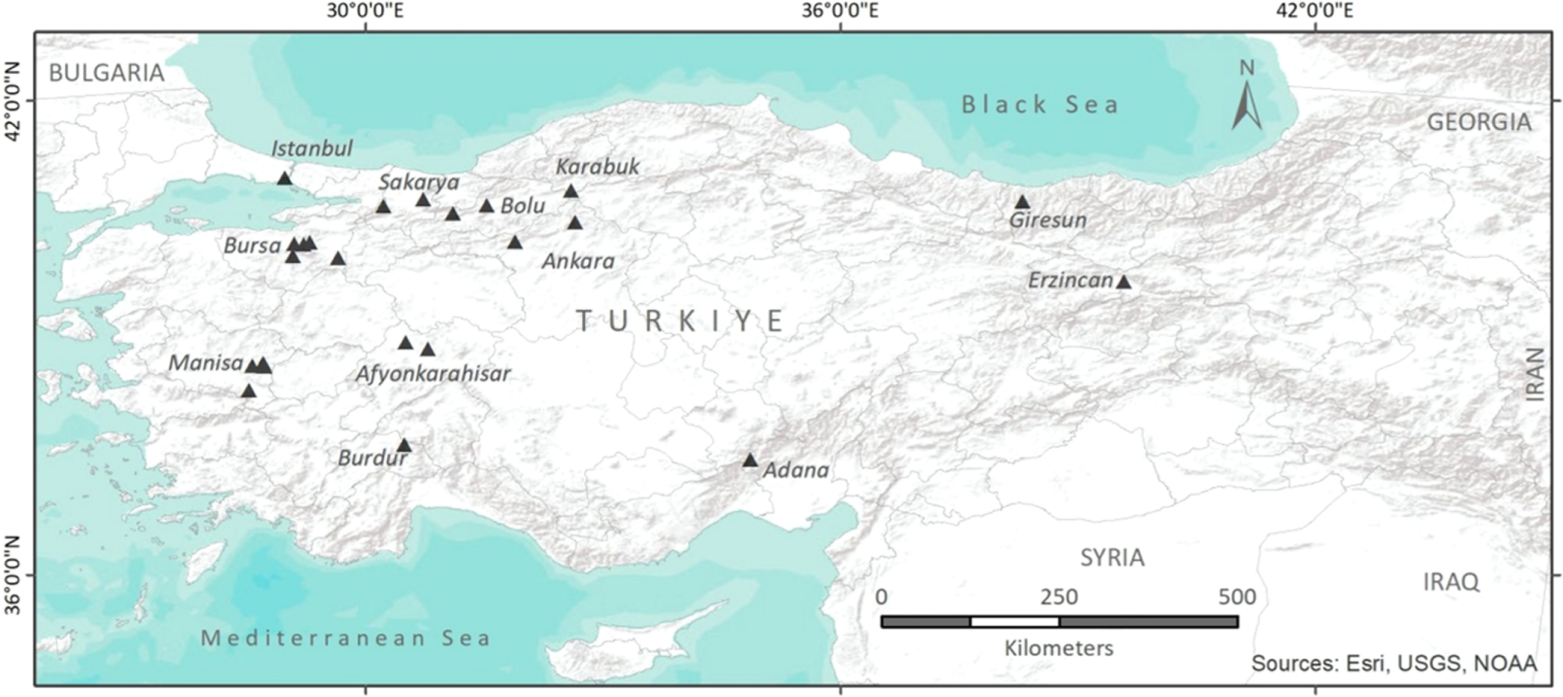
Locations of bottled mineral waters.

Currently, 30 companies approved by the Turkish Ministry of Health are bottling natural mineral water. In Turkey, most BMWs are sold in 0.2, 0.25, and 0.330 L volumes of metal screw-cap glass bottles.^14^ For this study, a total of 25 brands (23 of them carrying Turkish brand names and two being imported brands) commercially available in the bottled water sector were selected as the preferred popular brands throughout the country. The selected brands cover approximately 80% of the Turkish market. The origins of the BMW samples were geographically distributed across different regions of Turkey, as shown in Figure 1.^30^ In total, 58 bottled carbonated plain and fruit-flavored mineral water samples corresponding to these brands were purchased from markets in Turkey. These natural plain and fruit-flavored mineral water samples were coded as BPMW and BFMW to keep the brand names confidential, respectively.

For the γ-ray spectrometric measurements, each BMW sample was transferred to a polystyrene sample container whose geometry and size were the same as the reference source prepared for detector efficiency.^31^ Then, each sample container was tightly wrapped with Teflon tape to seal the radon (^222^Rn) gas. The BMW samples were kept for at least one month to achieve a secular equilibrium between ^226^Ra and ^222^Rn and ^228^Ra and ^228^Ac.^31^

### 2.2 Measurement of Radionuclide Concentrations

The activity concentrations of ^226^Ra,^228^Ra, and ^40^K in the BMW samples were measured using a γ-ray spectrometer with a p-type HPGe coaxial detector (ORTEC GEM50P4-83) with an energy resolution of 1.9 keV at a 1.33 MeV γ-ray line of ^60^Co and a relative efficiency of 50%.^32,33^ The detector is shielded with a cylindrical lead container of 10 cm to minimize the background radiation. It is connected to the detector interface module and a full-featured 16k multichannel digital spectrum analyzer with advanced digital signal processing. The full energy peak (FEP) efficiency calibration of the HPGe detector was performed using the standard solution prepared from natural uranium (RGU-1) purchased from the IAEA. The details of the procedures for the preparation of the standard solution are given in the study carried out by Kurnaz et al.^31^ The standard solution was placed on the detector and counted until good statistics. The γ-ray lines (photopeaks) of 63.3, 186.2, 295.2, 351.9, 609.3, and 1764.5 keV in equilibrium with ^226^Ra were used for the efficiency calibration of the detectors. The FEP efficiencies (ε_γ_) of these γ-ray lines were fitted as follows
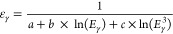
1where *E*_γ_ is the energy of the γ-ray photopeak and *a*, *b*, and *c* are 142.9, −40.3, and 0.5, respectively. Each BMW container was placed on the detector, and background measurements were counted for 50,000 s. Thus, the γ-ray spectrum of each BMW sample was obtained. γ Spectroscopy software (γ Vision 5.0) was used to evaluate the γ-ray spectrum (calculation of uncertainty of photopeaks, determination of radionuclides, measurement of uncertainty, etc.). The activity concentration of ^226^Ra was determined using the average activity concentrations of the weighted average concentrations of γ-ray lines from ^214^Pb (295.2 and 352.9 keV) and ^214^Bi (609.3 and 1764.5 keV). The activity concentration of ^228^Ra was determined using the weighted average concentrations of γ-ray lines from ^228^Ac (338.4 and 911.2 keV), while the activity concentration of ^40^K was measured directly by its γ-ray line of 1460.8 keV.^32^ The activity concentration (*A* in Bq/L) of each radionuclide was determined using the following equation
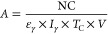
2where NC is the net count of γ-ray photopeak by subtracting the count of the γ-ray photopeak in the background spectrum, ε_γ_ is the efficiency of the γ-ray line given in eq 2, *I*_γ_ is the emission probability of the γ-ray line, *T*_C_ is the counting time (s), and *V* is the volume of the BMW sample (L). Standard solutions of potassium prepared from KCl (Merck) and KI (Sigma-Aldrich) standard solution and deionized water were utilized for validation of this method. The minimum detectable activity concentration (MDAC) for the γ-ray measurement system was calculated by the following equation.^34,35^
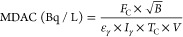
3where *F*_C_ is the statistical coverage factor equal to 1.64 (confidence level 95%) and *B* is the background counts over the region of interest for each radionuclide.^36^ The MDAC values for the radionuclides of interest were calculated as 0.2, 0.3, and 1.9 Bq/L for ^226^Ra,^228^Ra, and ^40^K, respectively.

The extended measurement uncertainty of the activity concentration (Δ*A*) was calculated using the following equation^36^
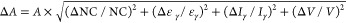
4where ΔNC is the count rate uncertainty, Δε_γ_ is the efficiency uncertainty, Δ*I*_γ_ is the emission probability uncertainty found in the nuclear data tables, and Δ*V* is the volume uncertainty.

### 2.3 Assessment of Radiological Risks

A consumer may be exposed to internal ionizing radiation emitted from the radionuclides in the ingested BMW.^11^ This radiological dose can be harmful with prolonged exposure, so it is important to estimate an individual’s annual effective ingestion dose based on the measured activity concentrations of the radionuclides. The radiological risk associated with ingestion of each BMW sample was assessed by estimating the annual effective ingestion dose and excess lifetime cancer risk. The AED (in μSv/y) was estimated using the following formula^31^
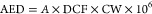
5where *A* is the activity concentration of the radionuclides (Bq/L), DCF is the dose conversion factor for ingestion, and CW is the annual consumption of BMW per capita (L/y). The DCF values for ^226^Ra,^228^Ra, and ^40^K are taken as 2.8 × 10^–7^, 6.9 × 10^–7^, and 6.2 × 10^–9^ Sv/Bq, respectively.^37^

The LCR of developing cancer, as a result of radionuclide intake through ingestion of BMW, was estimated using the following formula^5,38^
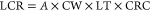
6where LT is the average lifetime (79 years) for adults^39^ and CRC is the cancer (mortality) risk coefficient for ingestion of the radionuclides in the BMW. The CRC values for ^226^Ra,^228^Ra, and ^40^K are taken as 7.17 × 10^–9^, 2.00 × 10^–8^, and 4.30 × 10^–10^ 1/Bq, respectively.^40^

## 3 Results and Discussion

### 3.1 Radionuclide Concentrations

**Figure 2:**
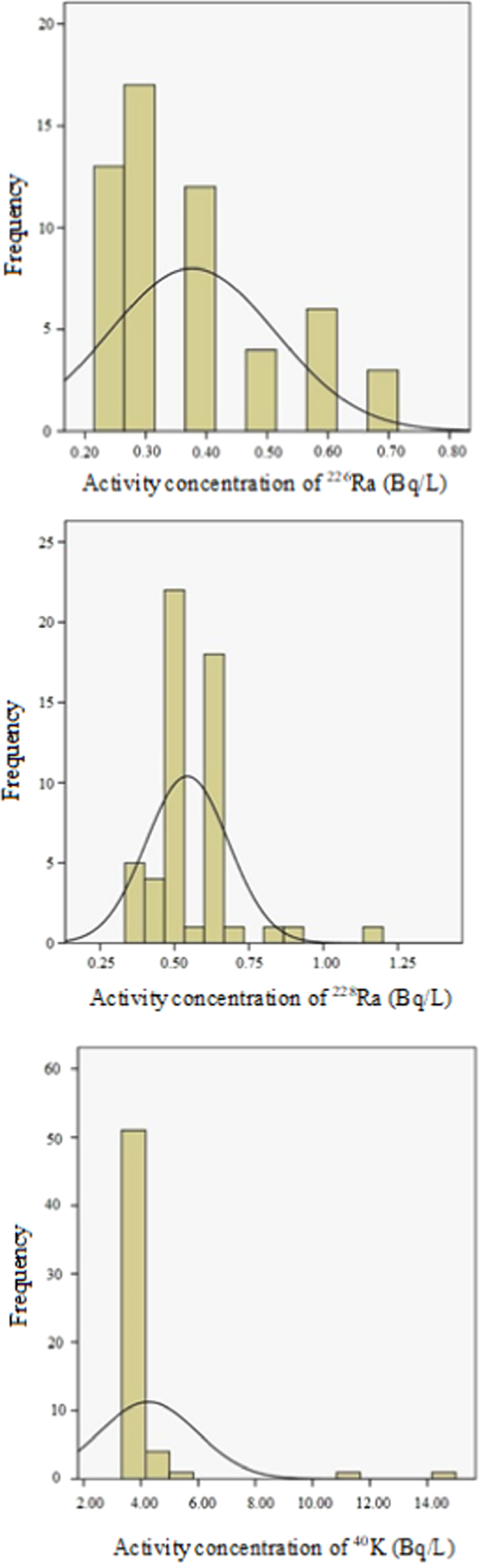
Frequency distributions of the activity concentrations of ^226^Ra,^228^Ra, and ^40^K in bottled mineral water samples.

Some descriptive statistical data (average, median, skewness, kurtosis, etc.) related to the activity concentrations of ^226^Ra,^228^Ra, and ^40^K measured in BMW samples are given in Table 1. The frequency distributions of these radionuclides are shown in Figure 2. Also, the distributions of these radionuclides in BPMW and BFMW samples are presented in Tables 2 and 3, respectively. It can be observed that the activity concentrations of ^226^Ra (half-life, 1600 years and α-ray emitter) and ^228^Ra (half-life, 5.75 years and β-ray emitter) measured in the investigated BMW samples varied from <MDA to 0.70 Bq/L with an average of 0.38 Bq/L and <MDA to 1.20 Bq/L with an average of 0.54 Bq/L. As can be seen from Figure 2, the frequency distributions of concentrations of ^226^Ra and ^228^Ra show the log-normal distribution. Approximately 72 and 84% of the activity concentrations of ^226^Ra and ^228^Ra measured in BMW samples are in the range of 0.24–0.40 Bq/L and 0.34–0.65 Bq/L, respectively. It can be observed that the activity concentrations of ^228^Ra are greater than those of ^226^Ra in most BMW samples. From Tables 2 and 3, the average activity concentrations of ^226^Ra measured in the BPMW and BFMW are found as 0.38 and 0.37 Bq/L, respectively. The highest ^226^Ra concentration is measured in BPMW11, BFMW5, and BFMW24 samples. The average activity concentrations of ^228^Ra measured in BPMW and BFMW are found as 0.51 and 0.57 Bq/L, respectively. The highest ^228^Ra concentration is measured in BFMW31 samples.

**Table 1:** Descriptive Statistical Data on the Activity Concentrations of Radionuclides Measured in Bottled Mineral Water Samples

	activity concentration (Bq/L)		
	^226^Ra	^228^Ra	^40^K
average	0.38	0.54	4.26
median	0.30	0.50	3.90
standard error	0.02	0.02	0.22
standard deviation	0.14	0.14	1.71
skewness	1.02	2.26	5.43
kurtosis	–0.06	9.19	30.01
minimum	<MDA	<MDA	3.80
maximum	0.70	1.20	14.80

**Table 2:** Radionuclide Concentrations Measured in Bottled Plain Mineral Water Samples

	activity concentration (Bq/L)		
sample code	^226^Ra	^228^Ra	^40^K
BPMW1	0.50 ± 0.15	0.50 ± 0.12	5.10 ± 0.30
BPMW2	0.40 ± 0.13	0.50 ± 0.12	3.90 ± 0.20
BPMW3	0.60 ± 0.14	0.60 ± 0.13	3.90 ± 0.20
BPMW4	0.30 ± 0.10	0.35 ± 0.10	3.80 ± 0.10
BPMW5	0.30 ± 0.10	0.60 ± 0.13	3.90 ± 0.10
BPMW6	0.30 ± 0.10	0.40 ± 0.11	3.80 ± 0.10
BPMW7	0.24 ± 0.05	0.80 ± 0.18	3.90 ± 0.10
BPMW8	0.30 ± 0.10	0.50 ± 0.12	4.10 ± 0.20
BPMW9	0.40 ± 0.10	0.60 ± 0.13	4.20 ± 0.20
BPMW10	0.25 ± 0.05	0.60 ± 0.13	3.80 ± 0.10
BPMW11	0.70 ± 0.10	0.50 ± 0.12	3.80 ± 0.10
BPMW12	0.25 ± 0.05	0.50 ± 0.12	3.90 ± 0.10
BPMW13	0.50 ± 0.16	0.60 ± 0.13	4.10 ± 0.20
BPMW14	<MDA	0.40 ± 0.11	3.90 ± 0.10
BPMW15	0.40 ± 0.12	0.50 ± 0.12	3.80 ± 0.10
BPMW16	<MDA	0.60 ± 0.13	3.80 ± 0.10
BPMW17	0.60 ± 0.14	<MDA	3.90 ± 0.10
BPMW18	0.26 ± 0.05	0.37 ± 0.10	4.00 ± 0.20
BPMW19	0.50 ± 0.12	0.50 ± 0.12	3.90 ± 0.10
BPMW20	0.27 ± 0.04	0.50 ± 0.12	4.10 ± 0.20
BPMW21	0.28 ± 0.05	0.40 ± 0.11	3.90 ± 0.10
BPMW22	0.29 ± 0.05	0.34 ± 0.10	3.90 ± 0.10

**Table 3:** Radionuclide Concentrations Measured in Bottled Fruit-Flavored Mineral Water Samples

	activity concentration (Bq/L)		
sample code	^226^Ra	^228^Ra	^40^K
BFMW1	0.60 ± 0.15	0.60 ± 0.13	3.90 ± 0.20
BFMW2	0.30 ± 0.10	0.50 ± 0.12	11.20 ± 0.30
BFMW3	0.25 ± 0.06	0.50 ± 0.10	14.80 ± 0.40
BFMW4	0.40 ± 0.10	0.90 ± 0.19	4.40 ± 0.20
BFMW5	0.70 ± 0.17	0.60 ± 0.13	3.80 ± 0.20
BFMW6	0.40 ± 0.12	0.60 ± 0.12	4.00 ± 0.20
BFMW7	0.24 ± 0.05	0.50 ± 0.11	4.40 ± 0.20
BFMW8	0.50 ± 0.12	0.60 ± 0.12	3.90 ± 0.10
BFMW9	0.60 ± 0.14	0.50 ± 0.11	3.90 ± 0.10
BFMW10	0.25 ± 0.06	0.50 ± 0.12	3.80 ± 0.20
BFMW11	0.26 ± 0.05	0.70 ± 0.16	3.90 ± 0.20
BFMW12	0.40 ± 0.12	0.60 ± 0.13	4.10 ± 0.20
BFMW13	0.40 ± 0.12	0.50 ± 0.12	3.80 ± 0.20
BFMW14	0.60 ± 0.13	<MDA	4.30 ± 0.20
BFMW15	0.30 ± 0.10	0.35 ± 0.10	3.80 ± 0.10
BFMW16	0.40 ± 0.12	0.60 ± 0.13	3.90 ± 0.10
BFMW17	0.40 ± 0.12	0.40 ± 0.10	3.90 ± 0.10
BFMW18	0.40 ± 0.12	0.50 ± 0.11	3.80 ± 0.10
BFMW19	0.60 ± 0.14	0.60 ± 0.13	3.90 ± 0.10
BFMW20	0.27 ± 0.05	0.50 ± 0.11	3.80 ± 0.10
BFMW21	0.24 ± 0.08	0.50 ± 0.12	4.10 ± 0.20
BFMW22	0.30 ± 0.10	<MDA	3.90 ± 0.10
BFMW23	0.24 ± 0.07	0.36 ± 0.10	3.80 ± 0.10
BFMW24	0.70 ± 0.17	0.60 ± 0.13	3.90 ± 0.10
BFMW25	0.40 ± 0.12	0.50 ± 0.11	4.10 ± 0.20
BFMW26	0.40 ± 0.12	0.50 ± 0.11	3.90 ± 0.10
BFMW27	0.24 ± 0.07	0.60 ± 0.12	3.80 ± 0.10
BFMW28	0.25 ± 0.07	0.60 ± 0.12	3.80 ± 0.10
BFMW29	0.30 ± 0.10	0.50 ± 0.11	3.90 ± 0.10
BFMW30	0.30 ± 0.10	0.50 ± 0.11	3.80 ± 0.10
BFMW31	0.30 ± 0.10	1.20 ± 0.20	3.80 ± 0.10
BFMW32	0.24 ± 0.05	<MDA	4.00 ± 0.20
BFMW33	0.30 ± 0.10	<MDA	3.80 ± 0.10
BFMW34	<MDA	0.60 ± 0.13	3.80 ± 0.10
BFMW35	0.30 ± 0.10	0.50 ± 0.12	4.10 ± 0.20
BFMW36	0.30 ± 0.10	0.60 ± 0.13	3.80 ± 0.10

The activity level of ^40^K (half-life, 1.28 × 10^9^ years and γ-ray emitter) in a healthy individual is kept constant by a range of physiological processes to regulate the functions of the body. Therefore, the levels of ^40^K generally were not considered in assessing radiological hazards to health caused by radionuclides in drinking water.^18,31^ The activity concentrations of ^40^K measured in the investigated BMW samples varied from 3.80 to 14.80 Bq/L with an average of 4.26 Bq/L. The frequency distribution of concentrations of ^40^K shows the log-normal distribution. The average activity concentrations of ^40^K measured in the BPMW and BFMW are found as 3.97 and 4.43 Bq/L, respectively. The highest ^40^K concentration was measured in BFMW3 samples.

Table 4 presents the comparison of the activity concentrations of the radionuclides in the BMW samples with those determined in previous studies in different countries and guidance levels recommended by the WHO and EPA for drinking water. As can be seen in Table 4, the activity concentrations of ^226^Ra are lower than those consumed in Iran, Malaysia, and Spain. Also, all activity concentrations of ^226^Ra are lower than the MPL of 1 Bq/L set by the WHO.^18^ The activity concentrations of ^228^Ra are lower than those consumed in Belarus, Iran, and Malaysia. All activity concentrations of ^228^Ra are higher than the MPL of 0.1 Bq/L set by the WHO.^18^ Also, activity concentrations of ^226^Ra and ^228^Ra above the MDA values are higher than the maximum contaminant level of 0.185 Bq/L set by the EPA.^47^ The activity concentrations of ^40^K are lower than those consumed in Belarus and Iran. Also, all activity concentrations of ^40^K, except for BFMW2 and BFMW3, are lower than the MPL of 10 Bq/L set by the WHO for drinking waters.^18^

**Table 4:** Comparison of the Activity Concentration of Radionuclides in Bottled Mineral Water Samples with the Literature Values and Guidance Levels

		activity concentration (Bq/L)			
country	N	^226^Ra	^228^Ra	^40^K	reference
Algeria	5 samples	0.013–0.1489	0.0072–0.0529	0.07–2.19	(41)
Austria	24 samples	0.002–0.211	0.005–0.236		(42)
Belarus	17 samples	<0.005–0.622	<0.01–2.08		(43)
Brasil	17 brands	<0.0022–0.647	0.012–0.741		(44)
Bulgaria	14 samples	0.025–0.211			(23)
Iran	70 samples	<0.03–3.88	0.013–13.75	1.29–389.17	(5)
Italy	21 samples	0.002–0.2			(45)
Malaysia	8 brands	1.46–3.30	0.65–3.39	21.12–25.31	(11)
Poland	30 samples	0.003–0.641	0.02–0.25		(10)
Romania	10 samples	0.029–0.45		0.14–1.28	(6)
Spain	30 brands	0.01–1.52			(46)
Tunisia	6 samples	0.002–0.067	0.002–0.030		(21)
Turkey	13 samples	0.061–0.267		0.108–1.404	(25)
Turkey	8 samples		0.1–1.04		(27)
Turkey	58 samples	<0.2–0.70	<0.3–1.20	3.80–14.80	this study
WHO	1	0.1	10	17	
EPA	0.185				

### 3.2 Risk Assessment

The annual effective doses and excess lifetime cancer risks due to the ingestion of BMWs were estimated for adults in two different scenarios according to the intake of the waters. In the first scenario, annual water consumption per capita was taken as the yearly consumption of BMW in Turkey (13 L/y).^15^ In the second scenario, annual water consumption per capita was taken as the yearly consumption of bottled drinking water in Turkey (150 L/y).^48^ The values of the AEDs and LCRs estimated for two scenarios are given in Table 5. As far as the measured activity concentrations of the radionuclides are concerned, the total AEDs for all of the investigated BMWs varied from 1.2 to 12.2 μSv/y with an average of 6.1 μSv/y for the first scenario and 13.8–140.3 μSv/y with an average of 70.3 μSv/y for the second scenario. The average contributions of ^226^Ra, ^228^Ra, and ^40^K to the total AEDs are 25, 68, and 7%, respectively. ^228^Ra, which is one of the most radiotoxic naturally occurring radionuclides, is the highest contributor to the total AEDs of all BMW samples. All total AEDs estimated for the first scenario are significantly lower than the guidance dose level or individual dose criterion of 100 μSv/y recommended by the WHO, Turkish legislation, and EU directive. For the second scenario, except for two samples BFMW4 (114 μSv/y) and BFMW31 (140 μSv/y), all total AEDs are below the quoted dose criterion. The total LCRs of all of the investigated BMWs estimated for the first and second scenarios varied from 3.5 × 10^–6^ to 2.9 × 10^–5^ with an average of 1.5 × 10^–5^ and 4.1 × 10^–5^ to 3.3 × 10^–4^ with an average of 1.7 × 10^–4^, respectively. All of the total LCR values are lower than the acceptable level of 10^–3^.^5,49^

**Table 5:** Annual Effective Doses Due to the Consumption of Bottled Mineral Water Samples

			annual effective dose (μSv/y)			
scenario	water type		^226^Ra	^228^Ra	^40^K	total
first	BPMW	average	1.4	4.6	0.3	6.2
		minimum	0.9	3.0	0.3	4.4
		maximum	2.5	7.2	0.4	8.4
	BFMW	average	1.4	5.1	0.4	6.2
		minimum	0.9	3.1	0.3	1.2
		maximum	2.5	10.8	1.2	12.2
	all BMW samples	average	1.4	4.9	0.3	6.1
		minimum	0.9	3.0	0.3	1.2
		maximum	2.5	10.8	1.2	12.2
second	BPMW	average	16.0	52.5	3.7	68.4
		minimum	10.1	35.2	3.5	28.8
		maximum	29.4	82.8	4.7	96.5
	BFMW	average	15.7	58.6	4.1	71.4
		minimum	10.1	36.2	3.5	13.8
		maximum	29.4	124.2	13.8	140.3
	All BMW samples	average	15.8	56.2	4.0	70.3
		minimum	10.1	35.2	3.5	13.8
		maximum	29.4	124.2	13.8	140.3

			lifetime cancer risk			
scenario	water type		^226^Ra	^228^Ra	^40^K	Total
first	BPMW	average	2.8 × 10^–6^	1.0 × 10^–5^	1.8 × 10^–6^	1.4 × 10^–5^
		minimum	1.8 × 10^–6^	7.0 × 10^–6^	1.7 × 10^–6^	6.1 × 10^–6^
		maximum	5.2 × 10^–6^	1.6 × 10^–5^	2.3 × 10^–6^	2.0 × 10^–5^
	BFMW	average	2.8 × 10^–6^	1.2 × 10^–5^	2.0 × 10^–6^	1.5 × 10^–5^
		minimum	1.8 × 10^–6^	7.2 × 10^–6^	1.7 × 10^–6^	3.5 × 10^–6^
		maximum	5.2 × 10^–6^	2.5 × 10^–5^	6.5 × 10^–6^	2.9 × 10^–5^
	all BMW samples	average	2.8 × 10^–6^	1.0 × 10^–5^	1.9 × 10^–6^	1.5 × 10^–5^
		minimum	1.8 × 10^–6^	7.0 × 10^–6^	1.7 × 10^–6^	3.5 × 10^–6^
		maximum	5.2 × 10^–6^	2.5 × 10^–5^	6.5 × 10^–6^	2.9 × 10^–5^
second	BPMW	average	3.2 × 10^–5^	1.2 × 10^–5^	2.0 × 10^–5^	1.6 × 10^–4^
		minimum	2.0 × 10^–5^	8.1 × 10^–5^	1.9 × 10^–5^	7.1 × 10^–5^
		maximum	5.9 × 10^–5^	1.9 × 10^–4^	2.6 × 10^–5^	2.3 × 10^–4^
	BFMW	average	3.2 × 10^–5^	1.3 × 10^–4^	2.3 × 10^–5^	1.7 × 10^–4^
		minimum	2.0 × 10^–5^	8.3 × 10^–5^	1.9 × 10^–5^	4.1 × 10^–5^
		maximum	5.9 × 10^–5^	2.8 × 10^–4^	7.5 × 10^–5^	3.3 × 10^–4^
	all BMW samples	average	3.2 × 10^–5^	1.3 × 10^–4^	2.2 × 10^–5^	1.7 × 10^–4^
		minimum	2.0 × 10^–5^	8.1 × 10^–5^	1.9 × 10^–5^	4.1 × 10^–5^
		maximum	5.9 × 10^–5^	2.8 × 10^–4^	7.5 × 10^–5^	3.3 × 10^–4^

## 4 Conclusions

The activity concentrations of ^226^Ra,^228^Ra, and ^40^K in 58 BMW samples of 25 different brands consumed in Turkey were determined using γ-ray spectrometry. Based on the measured activity concentrations of these radionuclides, the radiological health risks that may arise from ingestion of the investigated BMW samples were assessed for adults according to two different scenarios. The results revealed that the average ^228^Ra activity concentration measured in the investigated BMW samples was approximately five times higher than the WHO-recommended maximum allowable limit of 0.1 Bq/L for drinking water. The average total annual effective doses estimated for adults are lower than the WHO-recommended limit of 0.1 mSv/y for drinking water. However, the total annual effective doses of two BMW samples are above the quoted limit value. Also, all total excess lifetime cancer risks are below the acceptable level of 10^–3^. However, given the high radiotoxicity of ^228^Ra, its presence in BMW samples and the associated radiological health risk may require particular attention.

The data obtained in this study can contribute to the determination of the baseline levels of natural radioactivity in BMWs and provide basic information for consumers and competent authorities regarding the internal exposure risk due to the ingestion of the BMW. The ever-growing mineral water markets in Turkey make it important to ensure that the radioactivity levels in these BMWs are in line with the WHO-recommended level and are not expected to lead to health problems. Thus, these data can assist in the development of future regulations for the radiological protection of the Turkish population and be useful in working toward the assurance of the sale of safe BMWs.
